# Two is More Than One: How to Combine Brain Stimulation Rehabilitative Training for Functional Recovery?

**DOI:** 10.3389/fnsys.2015.00154

**Published:** 2015-11-10

**Authors:** Satoko Koganemaru, Hidenao Fukuyama, Tatsuya Mima

**Affiliations:** ^1^Brain Integrative Science, Kyoto University School of MedicineSakyo-ku, Kyoto, Japan; ^2^Human Brain Research Center, Kyoto University School of MedicineSakyo-ku, Kyoto, Japan

**Keywords:** use-dependent plasticity, associative plasticity, neuro-rehabilitation, transcranial magnetic stimulation (TMS), transcranial direct current stimulation (tDCS)

## Abstract

A number of studies have shown that non-invasive brain stimulation has an additional effect in combination with rehabilitative therapy to enhance functional recovery than either therapy alone. The combination enhances use-dependent plasticity induced by repetitive training. The neurophysiological mechanism of the effects of this combination is based on associative plasticity. However, these effects were not reported in all cases. We propose a list of possible strategies to achieve an effective association between rehabilitative training with brain stimulation for plasticity: (1) control of temporal aspect between stimulation and task execution; (2) the use of a shaped task for the combination; (3) the appropriate stimulation of neuronal circuits where use-dependent plastic changes occur; and (4) phase synchronization between rhythmically patterned brain stimulation and task-related patterned activities of neurons. To better utilize brain stimulation in neuro-rehabilitation, it is important to develop more effective techniques to combine them.

## Use-Dependent Plasticity Enhanced by the Combination of Rehabilitative Training and Brain Stimulation

Repetitive training is one of the fundamental strategies in neuro-rehabilitation regardless of what type of damage has occurred in the central or peripheral nervous system. Task-specific training induces task-specific neuronal changes lasting for a long period i.e., use-dependent plasticity that can lead to functional recovery (Butefisch et al., 1995; Nudo and Milliken, 1996; Nudo et al., 1996a,b; Hummelsheim, 1999; Masiero and Carraro, 2008; Richards et al., 2008; Dimyan and Cohen, 2011). Use-dependent plasticity induced by motor training has been demonstrated within the human primary motor cortex (M1). The long-term potentiation (LTP)-like changes in specific cortcospinal motoneurons were induced for the trained task after repetitive simple finger movements (Classen et al., 1998; Butefisch et al., 2000; Rossini and Pauri, 2000).

In recent decades, a number of studies have shown that non-invasive brain stimulation such as repetitive transcranial magnetic stimulation (rTMS) or transcranial direct current stimulation (tDCS) has an add-on effect in combination with rehabilitative therapy (Platz and Rothwell, 2010; Edwardson et al., 2013; Sandrini and Cohen, 2013; Floel, 2014). Furthermore, this combination may better enhance functional recovery in post-stroke patients, as compared with rehabilitation training alone, which may not sufficiently induce functional recovery (Khedr et al., 2005, 2010; Kim et al., 2006, 2010; Takeuchi et al., 2008; Chang et al., 2010, 2012; Emara et al., 2010; Koganemaru et al., 2010; Conforto et al., 2012; Meehan et al., 2011; Nair et al., 2011; Stagg and Nitsche, 2011; Wang et al., 2012; Hsu et al., 2013), especially in the chronic phase when it is difficult to produce plastic changes (Nakayama et al., 1994; Verheyden et al., 2008). A single intervention of brain stimulation alone without rehabilitative therapy seems to have limited effects on patients with mild motor symptoms (Hummel and Cohen, 2005; Koganemaru et al., 2010) and insufficient sustainability of effects (Takeuchi et al., 2005; Kim et al., 2009). Whereas the combination may enhance use-dependent plasticity induced by repetitive training.

## Associative Plasticity to Produce the Combination Effects

Although the exact neurophysiological mechanism of this combination effect is not known yet, it may be based on Hebbian associative plasticity (Hebb, 1949). For example, a post-synaptic neuron (A) receives low-frequency weak inputs from one pre-synaptic neuron (B) (the inputs themselves cannot induce LTP in a synaptic connection). Simultaneously, neuron (A) receives high-frequency weak inputs (the inputs themselves can induce LTP) from another pre-synaptic neuron (C). According to the Hebbian rule, LTP is also induced in the weak synaptic connection between neurons (A) and (B) as well as between neurons (A) and (C). A similar mechanism would work in the case of the combination of training with brain stimulation. Training alone may only produce a weak activation of neuronal circuits, which do not lead to long term changes. On the other hand, brain stimulation can induce LTP-like changes for synaptic strength in stimulated areas (Pascual-Leone et al., 1994; Hallett, 2000; Fritsch et al., 2010; Dayan et al., 2013; Karabanov et al., 2015). Therefore, simultaneous training with brain stimulation would enable weak synaptic connections to induce associative LTP-like effects through the Hebbian rule.

However, a recent study reported no additional effects of theta-burst stimulation (TBS) in combination with standardized rehabilitative therapy in chronic stroke patients. That might be possibly due to a failure to induce associative plasticity. Neuronal activities enhanced by TBS may not have been associated with task-specific neuronal activities produced by the rehabilitative therapy (Talelli et al., 2012).

We can speculate and make a list of possible factors that may have weakened the therapeutic effects of combined rehabilitation and brain stimulation:

1. Diversities in diseases, particularly the locations of lesions
  – The effects of facilitatory rTMS over M1 depended on lesion location in post-stroke hemiparetic patients. The deterioration of finger function was seen in the patients with cortical lesions, whereas improvement in finger function was seen in patients with subcortical lesions (Ameli et al., 2009).
2. Small sample size
  – The responses to brain stimulation show a large variability even if patients are similar in lesion location, severity of paresis and time after stroke onset in patients. Genetic factors are responsible for individual susceptibility to rTMS-induced plasticity (Cheeran et al., 2008).
3. Insufficient intensity and/or too short duration of the intervention
  – Patients with severe paresis show reduced or no motor evoked potentials (MEP) with TMS (Pennisi et al., 1999; Hendricks et al., 2002). If the intensity of brain stimulation is determined by the excitability of the healthy hemisphere, it may be too weak to induce plasticity in the affected hemisphere. Unless brain stimulation is repeated daily for days to weeks, its effects might not be sustainable (Khedr et al., 2009; Emara et al., 2010; Bolognini et al., 2011; Conforto et al., 2012; Edwardson et al., 2013).
4. Inappropriate affinity between rehabilitation task and brain stimulation modality
  – This will be discussed in detail in the following section.

Future clinical studies should give careful consideration to these factors. We have considered how effectively we can induce associative plasticity through the combination of training and brain stimulation.

## Effective Methods of Combining Rehabilitative Training and Brain Stimulation

### Control of the Temporal Aspect Between Stimulation and Task Execution

First, we should control the temporal aspect between stimulation and task execution. Spike timing-dependent associative plasticity has been proven in both animals (Hess and Donoghue, 1996; Hess et al., 1996; Egger et al., 1999) and humans (Stefan et al., 2000, 2002; Ueki et al., 2006; Koganemaru et al., 2009). Associative LTP occurs when a post-synaptic neuron fires less than 10–20 ms after a pre-synaptic neuron. Recently, we have demonstrated that associative plasticity is induced within human M1. The repetitive pairing of TMS and paired bihemispheric stimulation (PBS) applied at a time interval of 15 ms, produced an associative LTP-like effect within the targeted M1 and facilitated fine finger movements (Koganemaru et al., 2009). Furthermore, associative use-dependent plasticity has been demonstrated within human M1. Thabit et al. (2010) showed that associative LTP-like changes were induced by the repetitive paring of a unidirectional finger movement and a single TMS pulse over the contralateral M1 with a specific interval in healthy subjects. It resulted in a faster reaction in the trained direction. By decrease or increase of the interval, LTP-like effects can disappear or be reversed. Buetefisch et al. (2011) showed that the extensor-specific M1 reorganization was induced by robot-assisted training of paretic wrist extension combined with TMS over the ipsilesional M1 in a strict temporal relationship in chronic post-stroke patients. Particularly, a decrease of motor threshold and a shift of motor mapping for the extensor carpi ulnaris muscles, not the biceps muscles, were demonstrated in the combination therapy with TMS over the ipsilesional M1. The training alone and the simulation protocol with TMS over the contralesional M1 did not show those changes. The results suggest that temporal associative plasticity is induced specifically for the extensor-related activity in the ipsilesional M1. If we can associate brain stimulation and task execution with proper timing, a task-specific associative plasticity may be induced. There is a large variability in movement onset of the paretic limbs after neurological insults. The central motor conduction time is prolonged in stroke and other neurodegenerative diseases due to lesions in the corticospinal tract (Kaviraja and Robert, 2013). It is influenced by time after disease onset or disease progression. On the other hand, electromyogram (EMG) onsets are often variable since it is difficult to increase firing rate in central nervous diseases (Barnes, 1980). Therefore, when we do simultaneous TMS with training, we may as well be careful for both the timing for TMS and task execution.

### Use of a Shaped Task for the Combination

An effective rehabilitative approach should consist of various types of training pertaining to body movements in enriched environments that encourage patterns and combinations of movement for improving recovery. Different types of task-specific training help their effects transfer into actual activities of daily life in patients (Teasell et al., 2005). However, a specific task or tasks for specific movements are better combined with brain stimulation. If a task consists of various gross and precise movements, it may evoke conflicting neuronal activities such as inhibition and activation. Then, it may reduce the effect of brain stimulation. In recent animal studies, it was shown that training general gross movements inhibited recovery of skilled movements (Garcia-Alias et al., 2009). Neural competition for newly available neural resources may occur when multiple tasks are trained (Reinkensmeyer and Boninger, 2012). In clinical studies, task-specificity of training is more important than the intensity of training (Page, 2003; Bayona et al., 2005). The repetition of task-specific training produced long-lasting cortical reorganization and use-dependent plasticity specific to the areas that were activated during the trained movements in healthy subjects (Classen et al., 1998; Butefisch et al., 2000). In post-stroke patients, shaped task-specific training resulted in a better recovery of their paretic upper-limb function as compared with general training (Butefisch et al., 1995; Woldag et al., 2010).

Recently, we have investigated the effect of repetitive motor tasks in the paretic upper-limb combined with brain stimulation in post-stroke patients (Koganemaru et al., 2010). Patients with chronic stroke with moderate-to-severe hemiparesis often suffer from motor deficits associated with flexor hypertonia. A possible therapeutic strategy is to selectively induce use-dependent plasticity in the extensors to counteract the flexor hypertonia. However, the beneficial effects of training in chronic-phase patients are relatively limited due to resistance to induction of use-dependent plasticity in the chronic phase (Nakayama et al., 1994; Verheyden et al., 2008). When 5 Hz rTMS over the ipsilesional M1 was combined with extensor training assisted by electrical neuromuscular stimulation, the combined intervention resulted in an improvement of extensor movement with a reduction of flexor hypertonia, whereas neither of the single interventions alone demonstrated any improvements. The extensor-specific change in M1 was likely attributable to a functional recovery of the paretic upper limb (Koganemaru et al., 2010). Our study is an exemplary case showing the relevance of task selection combined with brain stimulation to enhance use-dependent plasticity for functional recovery.

### The Stimulation of Neuronal Circuits Where Use-Dependent Plastic Changes Occur

Stimulation should be given to neuronal circuits and brain areas where use-dependent plastic changes occur. Because use-dependent plasticity is task-specific, changed circuits and areas depend on what type of task was trained. For repetitive simple motor tasks, use-dependent plastic changes have been reported within M1 (Classen et al., 1998; Butefisch et al., 2000; Rossini and Pauri, 2000). However, it is unknown whether it occurs within M1 alone or in combination with the multi-regional functional reorganization of the motor-related brain network. If it occurs in a multi-regional brain network, other non-M1 regions would be the possible targets of stimulation.

Recently, we used neuroimaging to investigate whether use-dependent changes occurred in a multiregional brain network in chronic post-stroke patients (Koganemaru et al., 2015). In process of post-stroke recovery with rehabilitative training, neuroimaging studies demonstrated that multi-regional brain reorganization occurred in several motor-related regions, including bilateral M1, premotor cortices (PMC), cingulate motor cortex (CMC), basal ganglia, and cerebellum (Nelles et al., 2001; Carey et al., 2002; Johansen-Berg et al., 2002a; Jang et al., 2003, 2005; Ward et al., 2003a, 2004; Luft et al., 2004; Ward and Cohen, 2004). The over-activity of non-M1 regions such as bilateral PMC in the acute stage was progressively decreased with an improvement in motor performance of the hemiparetic limbs (Calautti et al., 2001; Small et al., 2002; Ward et al., 2003a). In the chronic stage, the magnitude of brain activity in the non-M1 regions was negatively correlated with clinical outcome (Ward et al., 2003a,b) and positively correlated with the extent of damage in the corticospinal system (Ward et al., 2006). The findings suggest that the compensatory mechanism of these regions may be due to insufficient motor recovery (Feydy et al., 2002; Ward et al., 2003b). Those patients with larger brain damage and poorer clinical recovery may rely on activity in secondary motor areas to drive residual hand function (Johansen-Berg et al., 2002b). If a combination therapy of a task-specific training and brain stimulation could restore the ipsilesional M1 function by use-dependent plasticity, compensatory drive from secondary motor areas would be changed in post-stroke patients.

**Figure 1:**
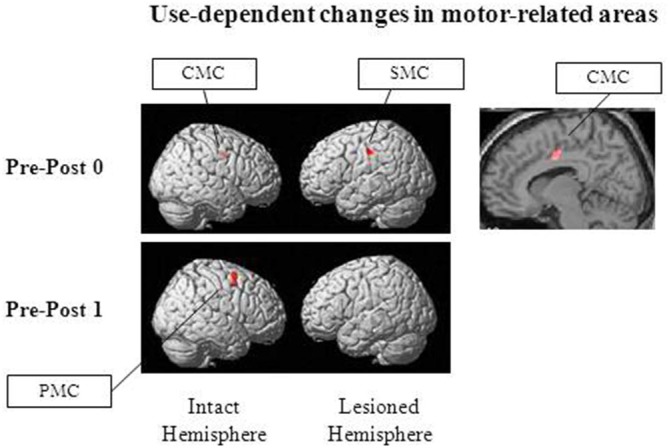
**Brain regions with use-dependent task-specific changes.** Reduced activation was observed in the ipsilesional SMC, the contralesional cingulate motor cortex (CMC) and the contralesional premotor cortices (PMC), specifically for extensor movements of the paretic upper-limb after our combination therapy consisting of 5 Hz repetitive transcranial magnetic stimulation (rTMS) and the paretic extensor training in post-stroke patients with flexor hypertonia (“Post 0” and “Post 1”, immediately after and 2 weeks after the combination therapy, respectively). Adapted from Koganemaru et al. (2015).

As previously described, we have developed a new combination therapy consisting of 5 Hz rTMS and an electrical neuromuscular stimulation assisted extensor training of the paretic upper-limb for stroke patients with flexor hypertonia. The extensor-specific plastic change in M1 was associated with beneficial functional effects (Koganemaru et al., 2010). We investigated whether extensor-specific multiregional brain reorganization occurred after our combination therapy by using functional magnetic resonance imaging (fMRI). The patients were scanned while performing upper-limb extensor movements. Untrained flexor movements were used as a control condition. Assessments were performed before, immediately after, and 2 weeks after the hybrid rehabilitation protocol. Analysis of the imaging data showed a significant reduction of brain activity in the ipsilesional SMC and the contralesional CMC immediately after (Post 0) and in the contralesional PMC 2 weeks after the intervention (Post 1; Figure 1). It suggests that the effects of the hybrid-rehabilitation appeared to differ temporally in each brain area. The process of motor learning consists of a fast learning stage and a slow learning stage. Specific neural representations are known in each stage (Karni et al., 1998; Kantak et al., 2012). The changes in activity in the ipsilesional SMC and the contralesional CMC may have shown combined effects of the fast learning stage, whereas the activity change in the contralesional PMC may have been involved in a consolidative process of the slow learning stage. Furthermore, the changes were associated with functional improvements of the paretic hands. They were not shown for the control condition (Koganemaru et al., 2015). Use-dependent plasticity induced by repetitive training may be related to the task-specific multi-regional brain reorganization. Thus, we expect that possible future targets for brain stimulation could include secondary motor areas. Artificial control of compensatory drive from secondary motor areas in accordance with the recovery process may be the next target for a combination therapy.

### Phase Synchronization Between Rhythmically Patterned Brain Stimulation and Task-Related Patterned Activities of Neurons

**Figure 2:**
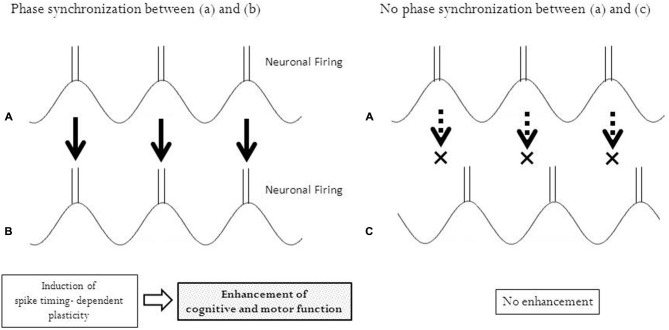
**Phase synchronization and temporal associative plasticity.** Phase synchronization enables neurons in two different regions **(A)** and **(B)** to fire at the same timing, leading to an induction of spike-timing dependent plasticity between these regions. No synchronization of phases between the two regions **(A)** and **(C)** with different timing of neuronal firing does not induce any plasticity.

In line with the theory of associative plasticity, we may be able to utilize the synchronization of phases between rhythmically patterned brain stimulation and task-related rhythmical activities of neurons. The phase synchronization of pre- and post-synaptic oscillations (wave-like neuronal signals) enabled researchers to correlate the timing of pre- and post-synaptic action potentials, resulting in the induction of temporal associative plasticity (Fell and Axmacher, 2011). Oscillatory non-invasive brain stimulation such as transcranial alternative current stimulation (tACS) and oscillatory tDCS (otDCS) has been reported to modulate oscillatory brain activity (Herrmann et al., 2013). Both tACS and otDCS use a sinusoidal form of electrical currents; however, tACS has no DC offset (net current = 0) and otDCS has a DC offset (net current = DC offset). In otDCS, the alternating current is superimposed onto a direct current. These protocols of stimulation may enhance neuronal circuits associated with intrinsic rhythmicity, leading to the enhancement of cognitive function (Marshall et al., 2006; Castro-Alamancos et al., 2007; Kanai et al., 2008, 2010; Kirov et al., 2009; Zaehle et al., 2010). In a recent study, phase-synchronized tACS suppressed Parkinson tremor by adjusting the phase to an abnormal cycle of the movements (Brittain et al., 2013). Rhythmical movements are produced with neuronal rhythmicity, a periodical repeat of excitation and inhibition. If oscillatory brain stimulation is synchronized with them at an appropriate phase, temporal associative plasticity may be induced (Figure 2).

One of the most familiar rhythmical movements in our daily life is locomotion, which requires the repeated patterned activation of specific neurons and muscles. Recently, in a preliminary experiment, we found that otDCS simulating gait rhythm induced gait-specific plasticity in healthy subjects (Koganemaru et al., 2014). Oscillatory patterned brain stimulation could be a new and powerful approach for the association of neuronal activities involved with training.

## Conclusion

We have proposed the possible strategies for combination therapy of stimulation and rehabilitative trainings: (1) the control of temporal aspect between stimulation and task execution; (2) the use of a shaped task for the combination; (3) the appropriate stimulation of neuronal circuits where use-dependent plastic changes occur; and (4) phase synchronization between rhythmically patterned brain stimulation and task-related patterned activities of neurons. Associative brain plasticity induced by the combination therapy can bring functional improvements in patients.

There are still many diseases that are resistant to neuro-rehabilitative approaches. To better utilize brain stimulation in neuro-rehabilitation, we must explore more effective techniques for combining brain stimulation and rehabilitative training. An efficient association between brain stimulation and rehabilitative training could improve brain plasticity and promote functional recovery of patients.

## Conflict of Interest Statement

The authors declare that the research was conducted in the absence of any commercial or financial relationships that could be construed as a potential conflict of interest.
